# Two Types of Laminoplasty for Cervical Spondylotic Myelopathy at Multiple Levels

**DOI:** 10.5402/2011/637185

**Published:** 2011-09-07

**Authors:** Shigeru Hirabayashi, Takashi Matsushita

**Affiliations:** Department of Orthopaedic Surgery, Teikyo University Hospital, 11-1, Kaga-2-Chome, Itabashi, Tokyo 173-8605, Japan

## Abstract

Based on the results from pathological analysis and computer simulations by means of finite element analysis that were reported before, the pathological changes of cervical spondylotic myelopathy (CSM) seem to begin at the posterolateral parts of the spinal cord, because the mechanical stress is mainly concentrated in these parts. With progression of the compression, the pathological changes become distributed to a wider area of the spinal cord. In patients with spinal canal stenosis, these changes spread to multiple levels of the cervical spine. Therefore, posterior decompression surgery at multiple levels such as cervical laminoplasty is thought to be reasonable.

## 1. Introduction

At present, the surgical methods of cervical laminoplasty are broadly divided into two types from the viewpoint of the site of osteotomy: open-door type and double-door type. Each method has its own advantages and disadvantages, and there is neither definite superiority nor definite inferiority between them. 

Postoperative C5 palsy is one of the problems to be resolved concerning cervical laminoplasty for CSM. At present, there are two theories concerning the cause of this palsy: (1) segmental spinal cord disorder and (2) nerve root injury. However, it is unclear yet which of these is correct. Based on our own anatomical analysis using 25 cadavers, we have concluded that this palsy is most likely caused by the C5 nerve root compression and stretch near the exit of the foramen. To prevent and decrease the postoperative C5 palsy, it is recommended that too severe lateral stretch of the multifidus muscles for a long time must be avoided. During laminoplasty, intermittent relaxation of tension of the hooks to the muscles may be one method of solution.

In this paper, the pathomechanism of cervical spondylotic myelopathy (CSM), the concept of the surgical treatment for CSM, the surgical procedures of two types of laminoplasty (open-door type and double-door type), and postoperative C5 palsy are reviewed mainly based on our own experiences.

## 2. Cervical Spondylosis

Cervical spondylotic myelopathy (CSM) is a disease from which the function of the cervical spinal cord is deteriorated by mainly compression from the surrounding skeletal structures due to various degenerative changes of the cervical spine with aging. These spondylotic changes are composed of structural changes at both anterior and posterior sides of the cervical spinal canal. 

At the anterior side of the spinal canal, narrowing of the intervertebral disc height with or without local instability, spur formation at the edge of the vertebral body, and formation of soft disc herniation appear. Ossification of the posterior longitudinal ligament (OPLL) is also one of these degenerative changes. At the posterior side of the spinal canal, buckling of hypertrophied flaval ligament and facet joint and its capsule into the spinal canal appears. Besides these changes at each vertebral level, the alignment of the whole cervical spinal column changes toward the kyphotic or hyperlordotic direction in some people. 

As a result, the spinal canal becomes progressively narrowed, and the spinal cord becomes gradually compressed. If the dynamic compression called “pincers mechanism” adds to these static compressions, the degree of its influence to the spinal cord becomes larger; that is, the spinal cord becomes severely compressed between the posterocaudal edge of the vertebral body and the lamina of the vertebra one level-below in extension position of the cervical spine. These spondylotic changes generally occur at the middle and lower levels of the cervical spine, usually in people of the age of 50 years and above.

## 3. Pathomechanism of Cervical Spondylotic Myelopathy

The cervical spinal cord, which is oval in shape, is situated in the cervical spinal canal which is triangularly shaped. Therefore, the bilateral posterolateral parts of the spinal cord are adjacent to the lamina and often contact with it, especially in patients with narrow spinal canal. The lateral corticospinal tracts within the white matter of the spinal cord are situated beneath these sites, and the primary afferent fibers enter into the spinal cord at these sites. 

With the progression of narrowing of the spinal canal due to cervical spondylosis, the spinal cord becomes gradually compressed and flattened. As the main motions of the human cervical spine are flexion and extension, compression force mainly applies to the spinal cord in the anteroposterior direction, which causes the compression and deformity of the spinal cord to progress. 

Ichihara et al. [1] presented computer simulations of the course of continuous compression applied to the spinal cord by means of finite element analysis. According to them, in combination with chronic anterior compression and acute posterior compression, the posterior aspect of the spinal cord is subjected to high shear stress. They suspect that in CSM, repeated hyperextension of the cervical spine results in intermittent acute compression of the spinal cord by buckling of the flaval ligament.

Wada and Yonenobu [2] presented the pathological changes within the spinal cord of cadavers with various severity of CSM in life. In mild cases, mild and localized demyelination of the lateral corticospinal tracts within the white matter was found. The gray matter was only flattened. In severe cases, extensive necrosis and gliosis in the lateral white column and central demyelination in the dorsal white columns were found. In the gray matter, extensive necrosis and cavitation were found. Even in severe cases, the structure of the anterior columns of the white matter was preserved.

After all, at the time of the initial phase of CSM, the mechanical stress is mainly concentrated in the posterolateral parts of the spinal cord. As a result, the pathological changes seem to begin at these sites. Because the alignment of the cervical spine is usually lordotic, the compression force to the spinal cord is thought to be larger at the posterior side than at the anterior one. This phenomenon is more obvious in patients with hyperlordotic cervical spine. The preservation of the anterior structure of the spinal cord, even in severe cases, supports this idea.

With the progression of the compression, the pathological changes become distributed to a wider area of the spinal cord. In patients with spinal canal stenosis, these changes spread to multiple levels of the cervical spine. This neurologic change worsens with additional disorders of blood supply to the spinal cord and finally reaches an irreversible state.

## 4. Concept of Surgical Treatment for CSM

Among various clinical courses of CSM, the candidates for surgery are patients whose conditions are chronically and progressively deteriorated despite various conservative treatments. It is obvious that the surgery must be performed before the pathological changes spread to the whole area of the cervical spine and toward an irreversible state although it is sometimes difficult to diagnose the condition precisely from neurological examinations. 

As the pathological changes of CSM are initiated at the posterolateral parts of the spinal cord, and the alignment of the cervical spine is usually lordotic, posterior decompression surgery is thought to be reasonable. As the main purpose of surgery is decompression of the spinal cord, the surgery must be performed at multiple levels in patients with wide range of compression.

There are two different surgical approaches for those patients: anterior and posterior.

Decompression surgery via anterior approach is usually performed for CSM with one or two level lesion without narrow spinal canal. Especially, it is useful for CSM accompanying radiculopathy. For rare CSM with severe kyphotic deformity at multiple levels for which posterior surgery has a limit to achieve adequate decompression, anterior surgery is also selected. However, in the anterior surgery, fixation using bone graft is mandatory after decompression. As a result, spinal instability at the adjacent levels may occur several years on. In CSM performed long fixation, dislocation of the grafted bone may occur soon after surgery.

Concerning the posterior approach, laminectomy has previously been performed as the main surgical method for CSM with multiple lesions in Japan. However, postoperative instability and the invasion of scar tissue into the spinal canal have often been recognized as severe complications after laminectomy. These complications were found to considerably relate to exaggeration of neurological conditions after surgery.

## 5. Cervical Laminoplasty

To decrease the complications of laminectomy, the idea of cervical laminoplasty was first introduced by Oyama and coworkers [3] under the name of “Expansive lamina-Z-plasty” in 1972. The concept of cervical laminoplasty was to decompress the multiple-level compression by preserving the posterior osseous structures of the cervical spine as much as possible and affording the opportunity for reactivation of the spinal cord in the widened spinal canal. Thereafter, various methods of cervical laminoplasty have been developed in Japan [4–8].

At present, the surgical methods of cervical laminoplasty are broadly divided into two types from the viewpoint of the site of osteotomy: these are, open-door type [4–6] and double-door type [7, 8]. In the open-door type, osteotomy is performed at one side of the lamina-facet junction. In the double-door type, osteotomy is performed at the central spinous process and lamina. Each method has its own advantages and disadvantages, and there is neither definite superiority nor definite inferiority between them.

## 6. Surgical Procedures of Cervical Laminoplasty

### 6.1. Open-Door Laminoplasty

After exposing the operated laminae, a longitudinal groove of 3 mm in width is made using an air drill along the lamina-facet junction line at the hinge side of the laminae, leaving the cancellous bone and the inner cortex undisturbed. Then, a similar groove is made at the open side, and finally, the inner cortex is severed.

**Figure 1:**
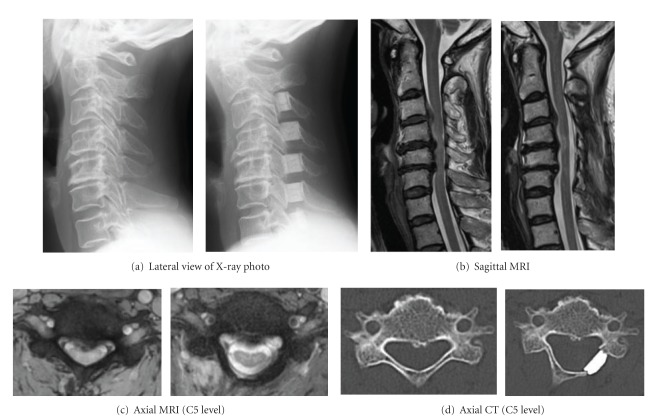
Various imaging findings of a 54-year-old female with CSM who underwent TBL at C3-C7 (left: before surgery, right: after surgery).

Next, the spinous process is pushed toward the hinge side by a finger. During this procedure, the hypertrophied flaval ligament at the open side is resected by a Kerrison punch. If hard resistance is felt, the groove in the hinge side is deepened slightly with the air drill. This procedure is repeated until the lamina is opened wide enough to insert a spacer of required width. In our standard method, a spacer of 12 mm in transverse length is fixed at the C5 and C6 levels. After hole-making procedures for thread fixation are completed, a spacer made of hydroxyapatite is fixed using a nonsoluble thread at each level (Figure 1).

### 6.2. Double-Door Laminoplasty

After exposing the operated laminae, the spinous processes are split centrally in order from the cranial to caudal levels using an air drill. At first, a triangle-shaped dome osteotomy is made at the cranial base of the spinous process to obtain a good visual field. Next, the remaining part of the spinous process and the inner plate of the lamina are split centrally. Then, a longitudinal groove of 3 mm in width is made bilaterally at the lamina-facet junction line taking care not to resect the inner cortex too deeply.

**Figure 2:**
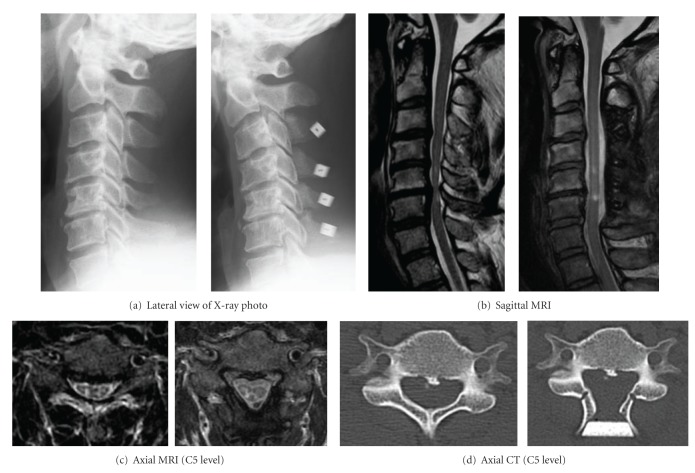
Various imaging findings of a 46-year-old male with CSM who underwent DDR at C3-C6 (left: before surgery, right: after surgery).

After opening the split spinous processes in a double-door fashion using a scissors, the constricting fibrous band above the dura mater and the hypertrophied flaval ligament are resected. At each level, the shape of the widened space is trapezoidal both on the axial and frontal sections. A hole to accommodate a thread for fixing the spacer is made at about 8 mm or more superficial from the inner plate of the lamina. The STSS spacer [9–15] whose shape is the same as that of the widened space and firmly stabilized is fixed using a nonsoluble thread at each level. In our standard method, an STSS spacer of 19 mm in cranial transverse length is fixed at the C5 and C6 levels (Figure 2). 

## 7. Comparison of the Widened Space in the Two Types of Laminoplasty

We compared the widened space of the cervical spinal canal at the C5 and C6 levels between two methods of cervical laminoplasty: tension-band laminoplasty (TBL, one method of open-door type) and double-door laminoplasty (DDR) [15]. The mean expansion ratio of the spinal canal was almost the same between TBL and DDL at the C5 level, however, it was significantly larger in TBL than DDL at the C6 level. The increase of inclination angle of the lamina was significantly larger in DDL than TBL at both the C5 and C6 levels.

## 8. Appropriate Indication of the Two Types of Laminoplasty

### 8.1. Respective Advantages and Disadvantages of TBL and DDL

It remains unclear to what minimal extent the spinal canal must be widened in order to obtain good surgical results. However, because the main purpose of the surgery is to decompress the neural structures, it is obvious that a wider spinal canal is preferable to obtain good results [16]. In TBL [6, 9, 15], the enlargement of the spinal canal depends only on the transverse length of a spacer. In contrast, in DDL, it depends on both the transverse length of the spacer and the depth of spacer fixation. Therefore, the control of enlargement of the spinal canal is easier in DDL than in TBL.

From the technical viewpoint, TBL has the advantage of being an easier decompression procedure than DDL, because two longitudinal grooves are made at the bilateral lamina-facet junction line. However, it is slightly more difficult in TBL to fix a spacer using a thread between the lamina and the inner edge of the facet joint. Also, the pathological findings at the hinge side cannot be visualized in TBL. And the biggest disadvantage of TBL is that the postoperative posterior skeletal structure of the cervical spine becomes unsymmetrical.

In contrast, DDL [8–15] has the advantages over TBL of easier fixation of spacers and direct visual confirmation of bilateral decompression, and the postoperative posterior skeletal structure of the cervical spine remains symmetrical. However, the procedures of making two longitudinal grooves at the bilateral lamina-facet junction line and one central split just above the dorsal surface of the dura mater must be performed. Also, the central split is slightly more difficult to perform in patients with large prominence of OPLL.

### 8.2. Appropriate Surgical Indications

It is thought that appropriate surgical indications of TBL are: (1) CSM combined with hemilateral radiculopathy, (2) large prominence of OPLL, and (3) patients with tiny spinous processes who cannot undergo DDL. From the viewpoint that the postoperative posterior skeletal structure of the cervical spine remains symmetrical in DDL, it is thought that appropriate surgical indications of DDL are (1) usual CSM, (2) small or slight prominence of OPLL, (3) CSM combined with bilateral radiculopathy, and (4) cervical canal stenosis combined with instability necessitating posterior spinal instrumentation surgery such as rheumatoid cervical spine. Posterior spinal instruments can be set bilaterally without being interrupted by inclined spinous processes [14].

## 9. Postoperative C5 Palsy in Cervical Laminoplasty

Even now, there remain several problems to be resolved concerning cervical laminoplasty for CSM: indication for CSM with moderate or severe kyphosis of the cervical spine, postoperative axial pain, and postoperative C5 palsy. 

In this paper, only the postoperative C5 palsy is presented and discussed based on our own anatomical analysis [17].

 Postoperative C5 palsy is defined as de novo muscle weakness at mainly the C5 lesion with slight or without sensory disturbance after cervical surgery. According to Sakaura et al. [18], who conducted literature review of 343 patients with C5 palsy, it was proved to have features as follows: (1) one-half of the patients were accompanied by sensory disturbance or intolerable pain at the C5 lesion. The other half of patients had only muscle weakness and no pain anywhere, (2) ninety-two percent of patients had hemilateral palsy and only 8 percent had bilateral palsy, (3) almost all palsy occurred within a week after surgery, and in rare patients, it occurred 2 or 4 weeks later, and (4) in rare patients, palsy occurred at the C6, C7, and C8 lesion alone or combined.

 At present, there are two theories concerning the cause of this palsy: (1) segmental spinal cord disorder and (2) nerve root injury. However, it is unclear yet which of these is correct. Based on our own anatomical analysis using 25 cadavers, we have concluded that this palsy is most likely caused by the C5 nerve root compression and stretch near the exit of the foramen.

**Figure 3:**
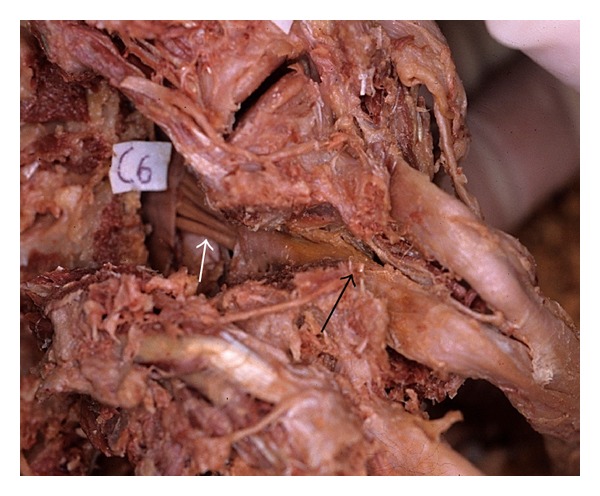
The anterior rootlet in the foramen (the facet joint is partially resected. Sight from a posterolateral side). The anterior rootlet (white arrow) runs adjacent to the tip of the superior facet (black arrow), where the foramen is narrowest.

**Figure 4:**
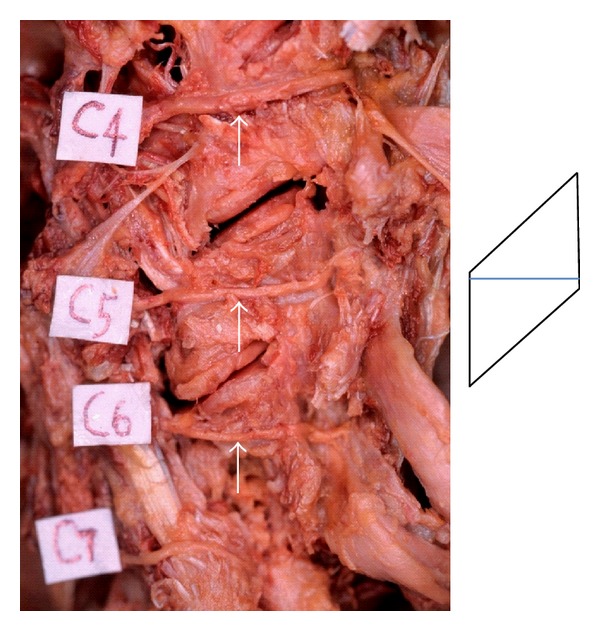
The running course of the medial branch of the posterior ramus (sight from a lateral side) and its schema. The medial branch of the posterior ramus (white arrow) runs posteriorly in the shortest distance of the line between the cranial side of the posterior tubercle of the transverse process and the posterior capsule of the facet joint in contact with the lateral side of the facet joint column.

 The results of our anatomical analysis concerning the cervical nerves were as follows: (1) among the cervical nerve roots composed of the brachial plexus, the distance between the division from the dura mater and the exit of the foramen is shortest at the C5 nerve root, (2) the anterior rootlet of the cervical nerve enters into the foramen anterocaudal side of the posterior rootlet and runs adjacent to the tip of the superior facet, where the foramen is narrowest (Figure 3), and (3) the cervical nerve is divided into the anterior and posterior rami. After dividing from the posterior ramus proper near the exit of the foramen, the medial branch of the posterior ramus runs posteriorly close to the cranial side of the posterior tubercle of the transverse process. It runs more posteriorly in contact with the lateral side of the facet joint column and finally divides into the capsule branch of the facet joint and the muscle branch of the multifidus muscles. After all, the medial branch of the posterior ramus runs in the shortest distance of the line between the cranial side of the posterior tubercle of the transverse process and the posterior capsule of the facet joint. This line corresponds to a diagonal line of a parallelogram that is formed at the lateral side of the facet joint column (Figure 4).

**Figure 5:**
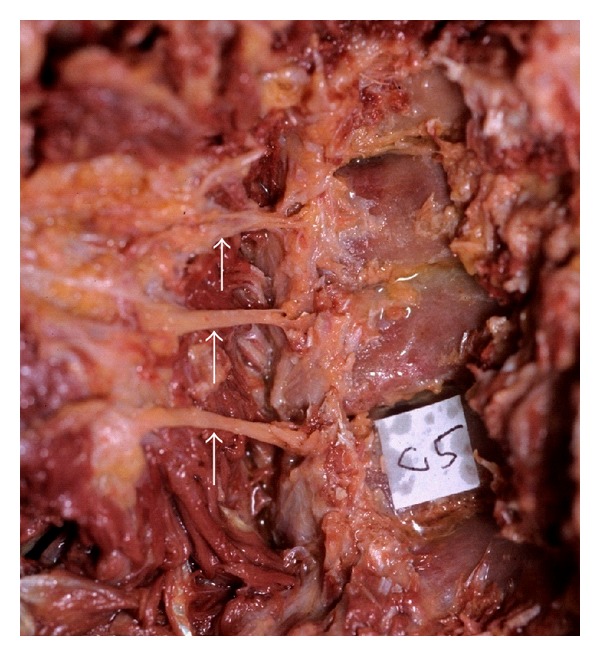
The muscle branch (white arrow) of the multifidus muscles of the medial branch of the posterior ramus (sight from a posterior side).

**Figure 6:**
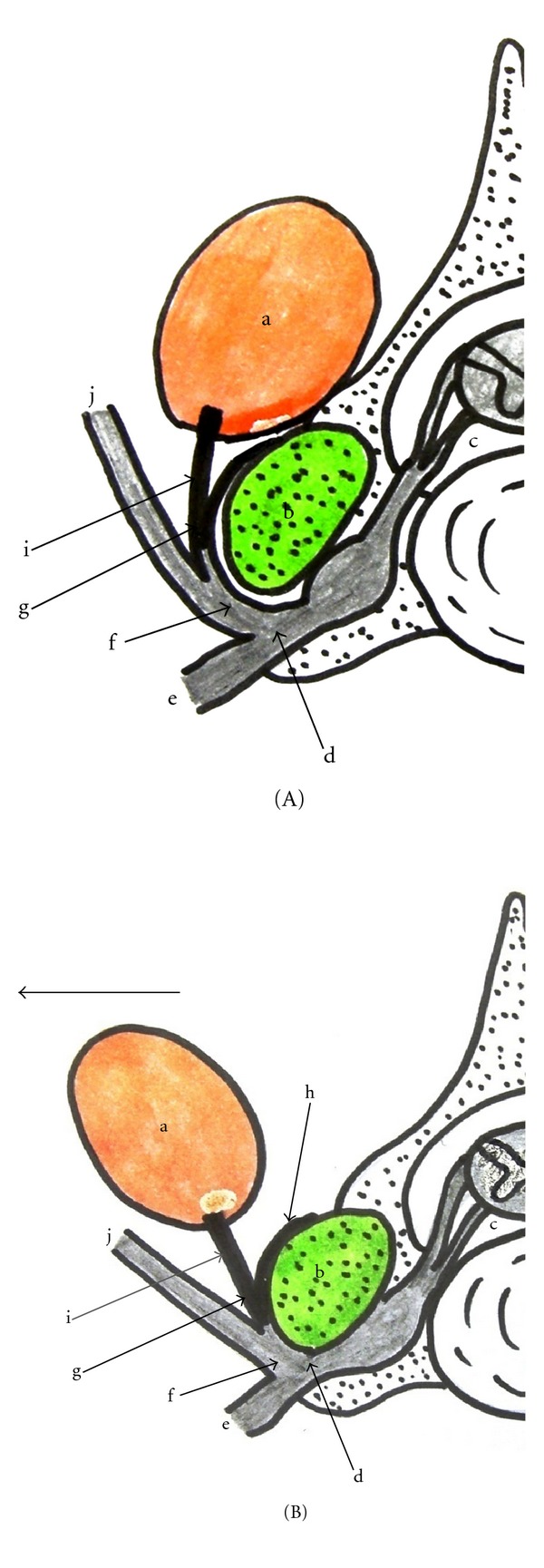
Schemata of a transverse section of the cervical spine. (A) Anatomical structure (B) condition of the multifidus muscle severely stretched laterally a: multifidus muscle, b: facet joint, c: anterior rootlet, d: spinal nerve, e: anterior ramus, f: posterior ramus, g: medial branch of the posterior ramus, h: capsule branch of the medial branch of the posterior ramus, i: muscle branch of the medial branch of the posterior ramus, and j: lateral branch of the posterior ramus. If the muscle branch of the multifidus muscles of the medial branch of the posterior ramus is severely stretched laterally, not only the medial branch of the posterior ramus, but also the anterior and posterior rami and the anterior rootlet are simultaneously stretched and compressed against adjacent structures such as hypertrophied facet joint.

Based on our anatomical analysis, the anterior rootlets of the cervical nerves seem to tend to mechanically be stretched and compressed in the foramen. If the muscle branch of the multifidus muscles of the medial branch of the posterior ramus (Figure 5) is severely stretched laterally during posterior surgery such as cervical laminoplasty, not only the medial branch of the posterior ramus but also the anterior and posterior rami and the anterior rootlet are simultaneously stretched and compressed against adjacent structures. This effect is thought to be largest in the C5 nerve root, because the running distance there is shortest and, therefore, the degree of free movement is most limited. In patients with hypertrophied facet joint due to degenerative changes, the influence of these stretches and compressions becomes larger, because the medial branch of the posterior ramus runs posteriorly in contact with the lateral side of the facet joint column (Figure 6).

 In the theory of nerve root injury, several features of postoperative C5 palsy can be well explained. The result that a half of the patients were accompanied by sensory disturbance or intolerable pain at the C5 lesion probably means that the C5 posterior rootlet is being simultaneously stretched or compressed. The result that 92% of patients had hemilateral palsy is well compatible with nerve root injury. However, hemilateral palsy of localized area (C5) in such a high percentage can almost never occur in segmental cord disorder. The result that almost all palsy occurred within a week after surgery means that the injury may have occurred during surgery or around the time of beginning rehabilitation. At the beginning of rehabilitation with the neck and head supported in an upright position after surgery, somewhat new mechanical stresses may concentrate in the spinal nerve, especially in the C5 nerve root due to the reasons mentioned above. 

 Even now, the exact cause of postoperative C5 palsy remains unclear. However, to prevent and decrease the postoperative C5 palsy, it is recommended that too severe lateral stretch of the multifidus muscles for a long time must be avoided. During laminoplasty, intermittent relaxation of tension of the hooks to the muscles may be one method of solution.
